# Motivational interviewing—an evidence-based, collaborative, goal-oriented communication approach in lifestyle medicine: A comprehensive review of the literature

**DOI:** 10.1016/j.jtumed.2023.03.011

**Published:** 2023-04-05

**Authors:** Mohammed Almansour, Sarah Ibrahim AlQurmalah, Habeeb Ibrahim Abdul Razack

**Affiliations:** a Department of Medical Education, College of Medicine, King Saud University, Riyadh, KSA; b Department of Family Medicine, Prince Sultan Military Medical City, Riyadh, KSA; c Faculty of Medicine & Health Sciences, Universiti Putra Malaysia, Serdang, Selangor, Malaysia; d Department of Cardiac Sciences, College of Medicine, King Saud University, Riyadh, KSA

**Keywords:** المقابلات التحفيزية((MI, وطب نمط الحياة( (LM, وتغيير السلوك, وتعاطي المخدرات, والنظام الغذائي, والنشاط البدني, Behavioral change, Diet, Lifestyle medicine, Motivational interviewing, Physical activity, Substance use

## Abstract

The global threat of noncommunicable diseases (NCDs) is alarmingly increasing. The health and economic burden of improper lifestyle choices is immense. Reducing modifiable risk factors has been demonstrated to significantly prevent chronic diseases. At this crucial time, lifestyle medicine (LM) has been recognized as an evidence-based medical domain applicable to NCDs. Among the tools used in LM, motivational interviewing (MI) is a patient-centered, collaborative counseling approach. In this evidence-based review article, we discuss recent literature on the application of MI in the six LM pillars defined by the British Society of LM (BSLM): healthy eating, mental wellbeing, healthy relationships, physical activity, minimizing harmful substances, and sleep. MI helps strengthen patients’ motivation to ameliorate behaviorally influenced health problems, improve treatment adherence, and optimize medical interventions. Technically correct, theoretically congruent, and psychometrically sound MI interventions yield satisfactory outcomes and help improve patient quality of life. Lifestyle change is often a gradual process involving multiple efforts and setbacks. MI is based on the idea that change is a process rather than an event. Extensive literature evidence supports the benefits of MI treatment, and interest in research on MI application is increasing across all BSLM pillars. MI helps people alter their thoughts and feelings about making changes by recognizing obstacles to change. Even interventions of short duration have been reported to yield better outcomes. Healthcare professionals must understand the relevance and importance of MI in clinical practice.

## الملخص

يتزايد التهديد العالمي للأمراض غير المعدية (NCDs) بشكل مثير للقلق. العبء الصحي والاقتصادي لاختيارات نمط الحياة غير الصحي هائل. ثبت أن الحد من عوامل الخطر القابلة للتعديل يمنع الأمراض المزمنة بشكل كبير. في هذا الوقت الحرج ، تلقى طب نمط الحياة (LM) اعترافًا جديدًا كمجال طبي قائم على الأدلة. من بين الأدوات المختلفة المستخدمة في طب نمط الحياة ، تأتي المقابلات التحفيزية (MI) كنهج استشاري تشاركي يركز على المريض. في هذه المقالة المستندة إلى الأدلة ، حاولنا إلقاء الضوء على الأدبيات الحديثة حول تطبيق MI على ركائز LM الستة التي حددتها الجمعية البريطانية لـطب نمط الحياة(LM (BSLM): الأكل الصحي ، والرفاهية العقلية ، والعلاقات الصحية ، والنشاط البدني ، التقليل من المواد الضارة والنوم الصحي. يساعد نهج (MI) في تقوية الجوانب التحفيزية للمرضى لمحاربة المشاكل الصحية المتأثرة بالسلوك ، وتحسين الالتزام بالعلاج ، وتحسين التدخلات الطبية. تحقق تدخلات MI الصحيحة من الناحية الفنية والمتطابقة نظريًا والسليمة من الناحية النفسية نتائج مرضية وتساعد على تحسين نوعية حياة المرضى. غالبًا ما يكون تغيير نمط الحياة عملية تدريجية تنطوي على جهود ونكسات متعددة. يعمل نهج MI على أساس أن التغيير عملية وليس حدثًا. الأدلة الأدبية حول فوائد نهج MI واسعة ، والبحث حول تطبيق MI يكتسب اهتمامًا عبر جميع ركائز طب نمط الحياة. تخدم MI الأشخاص لتغيير أفكارهم ومشاعرهم حول إجراء تغيير من خلال التعرف على ما يمنعهم من بدء عملية التغيير. حتى التدخلات ذات المدة الأقصر أثبتت قدرتها على تحقيق نتائج أفضل. يجب أن يتعلم المتخصصون في الرعاية الصحية أهمية وارتباط MI في الممارسة السريرية.

## Introduction

In 2015, the United Nations issued the 2030 Agenda for Sustainable Development, an integrated call for action in various human-centric global issues. Noncommunicable diseases (NCDs) are recognized as severe hazards among various health-associated areas of focus.^1^ Alarmingly, approximately 74% of all deaths reported globally are associated with NCDs. The World Health Organization (WHO) has warned that more than 75% of NCD-attributable deaths are reported in low- and middle-income countries.^2^ Direct and indirect expenses due to NCDs significantly affect productivity and human resources, and increase spending on diseases and illnesses. The five prominent NCDs of cardiovascular disease, chronic respiratory disease, cancer, diabetes, and mental disorders have been estimated to account for a financial burden of 47 trillion United States (US) dollars (US$; mean = US$ 2 trillion/year) between 2010 and 2030.^3^ Inappropriate lifestyle choices are key contributors to the development of NCDs. Thus, most chronic conditions are preventable. Reducing modifiable risk factors has been demonstrated to significantly prevent chronic diseases.

During this era of increasing NCD trends, lifestyle medicine (LM) has been recognized as an evidence-based medical domain. LM is not new but instead is the basis of age-old conventional medicine. The British Society of LM (BSLM) identifies six interlinked pillars of LM: healthy eating, mental wellbeing, healthy relationships, physical activity (PA), minimizing harmful substances, and sleep.^4^ The three key principles of LM are acknowledging the necessity of acting upon socioeconomic health factors, using evidence-based strategies to achieve sustained positive lifestyle changes, and having acquittance of the significance of the six LM pillars, with an understanding that no pillars exist in isolation. LM practitioners apply behavioral science concepts, value patients’ choices, and equip them with skills to create long-term behavioral changes.^4^

Among the various tools used in LM, motivational interviewing (MI) is a patient-centered, collaborative counseling approach. MI is “a client-centered, directive method for enhancing intrinsic motivation to change by exploring and resolving ambivalence.”^5^ It relies on autonomy, partnership, evocation, compassion, acceptance, and affirmation.^4^ The most effective MI practitioners are whole-hearted, empathic, pleasing, and directive in their communication with patients.^6^ MI helps strengthen patients' motivation to overcome behaviorally influenced health problems, improve treatment adherence, and optimize medical interventions.^7^ Technically correct, theoretically congruent, and psychometrically sound MI interventions yield satisfactory outcomes and help improve patients’ quality of life (QoL).^8^^,^^9^

In the present work, we review and discuss recent evidence of the application of MI in the six LM pillars defined by the BSLM. We searched PubMed and Google Scholar for scholarly articles (of any type or format) on the application of MI in different LM domains, published until November 8, 2022 in English, by using a combination of relevant keywords (motivational interviewing, lifestyle medicine, behavioral change, healthy eating, diet, nutrition, mental wellbeing/health, cognitive function, psychological wellbeing, mental disorders/illness, emotional wellness, mindfulness, loneliness, suicidal ideation, anxiety disorder, healthy relationships, PA, physical inactivity [PI], physical health, sedentary behavior, harmful substances, alcohol, recreational drugs, substance/drug abuse, tobacco, smoking, cannabis, sleep, sleep disorders, insomnia, obstructive sleep apnea, NCDs, obesity, weight control/management, type-2 diabetes mellitus [T2DM], and coronavirus disease 2019 [COVID-19]). Furthermore, we also reviewed the official websites of professional societies, such as BSLM and global organizations such as the United Nations, NCD Alliance, and WHO for relevant reports.

## Healthy eating

Eating is directly associated with health: a diet comprising unhealthful ingredients and ultra-processed food increases the risk of chronic diseases. LM specialists must advocate for MI interventions to address changes in eating patterns, respect and recognize patient choices, and follow a non-judgmental approach.^4^ In a recent two-arm randomized controlled trial (RCT), adult Dutch participants who intensively used the MyLifestyleCoach intervention, which was developed on the basis of MI and Self-Determination Theory, by following more sessions in the diet module, showed a greater increase in fruit and vegetable intake at 6 months, and a greater decline in the intake of unhealthful food at 12 months.^10^ In a 2-year efficacy study of combined MI and nutrition intervention, African women with T2DM (n = 12) showed improvements in diabetes-associated clinical and dietary self-care outcomes, such as the frequency of eating high-fat food and spacing carbohydrates throughout the day.^11^ MI-based interventions are cost-effective and results-oriented in this critical domain, and may even help promote healthful food among children.^12^ Interestingly, Smriti et al. have developed a Motivational Interviewing Conversational Agent to support in behavioral change in eating patterns among children. The findings support the implementation of MI-based innovative approaches.^13^ The LIFT RCT is aimed at evaluating dietary adherence intervention by assessing nutrient density (diet quality) and recommending food groups/eating patterns for patients with systematic lupus erythematosus. Patient enrolment is expected to be completed by June 2023.^14^

The World Obesity Atlas 2022 predicts that 1 billion people will live with obesity by 2030.^15^ MI approaches, used either individually or in combination with other techniques, are increasingly gaining reputation in weight and T2DM control programs. A cost-effective analysis of childhood obesity has reported an MI incremental cost-effectiveness ratio of $363 (savings: $3159) per child per percentile point reduction in body mass index for 2 years.^12^ A systematic review of RCTs (n = 21 studies) has found that MI-based telehealth intervention is effective in improving glycated hemoglobin, systolic blood pressure, T2DM self-efficacy, and PA behaviors.^16^ In another recent systematic review of adolescents (n = 19 studies), the MI intervention group showed a reduction in sugary beverage intake (standardized mean difference [SMD] = −0.47; K = 3; I^2^ = 26.2%) and waist circumference (SMD = −0.51; 95% confidence interval [CI] = −0.91, −0.11).^17^ In a meta-analysis (n = 10 studies) by Suire et al., MI interventions have been found to support weight management among women by producing significant changes in anthropometric outcomes. The effect sizes of MI in reducing body weight and body mass index were 0.19 (95% CI = −0.13, 0.26; p < 0.01) and 0.35 (95% CI = 0.12, 0.58; p < 0.01), respectively.^18^

## Mental wellbeing

According to the 2022 WHO World Mental Health Report, one in every six years lived with disability is attributed to mental disorders.^19^ Societal and environmental factors that may contribute to impaired mental health include feelings of hopelessness, cultural loss, racism, prejudice, discrimination, social polarization, poverty, economic downturns, disparity/inequality, public health emergencies, inadequate basic living amenities, humanitarian emergencies, long-term stress, forced displacement, social injustice, climate crisis, and pandemic threats.^4^^,^^19^ COVID-19 has negatively affected people's psychological wellbeing because of various factors, including loneliness, quarantine/confinement, movement restriction, grief, and loss. The Royal College of Psychiatrists warned of a “tsunami of mental illness” during the COVID-19 pandemic.^4^

Emotional wellness is a state of feeling good about oneself and having a positive outlook on life. Emotional wellbeing is central to having a positive approach to mental health. Mindfulness focuses on the present moment while accepting one's thoughts and emotions without judgment or criticism. Mindfulness helps reduce stress and improve overall QoL. MI can be an effective tool for emotional wellness and mindfulness, because it can help identify personal goals and increase insight and self-awareness. It also promotes engagement in active listening, which can help build rapport and trust with patients. MI focuses on the present and future rather than the past.^20^

In patients with severe mental illness (n = 1267) included in a 2019 meta-analysis, MI-based adherence therapy improved psychiatric symptoms (effect size = 0.45), and longer MI sessions and higher intervention doses showed superior effect sizes.^21^ Recovery and discharge plans in these patients followed a multifactorial process and involved efforts to streamline aftercare procedures. In a study by Kisely et al., the identification of triggers associated with recovery plans increased from 52% to 94% in the MI intervention group (n = 100 wards; χ^2^ = 23.3; df = 1; p < 0.001), inpatient input increased, and significant progress was observed regarding participant experiences in discharge planning.^22^ In a quasi-experimental study published in 2020, MI intervention in individuals with suicide attempts resulted in promising behavioral changes. Patients in the intervention group (n = 35) had a lower Beck Suicidal Scale Ideation score than controls (8.86 ± 5.30 vs. 15.85 ± 6.65; p = 0.0001). MI intervention also increased the patients’ rate of using mental health services (88.58% vs. 45.72%; p = 0.001).^23^

## Healthy relationships

Healthy and meaningful relationships, together with social connectedness, are crucial for optimal mental and physical health. Overcoming loneliness and reducing social isolation are essential components of LM.^4^ Older adults and patients with social anxiety disorder (SAD) face challenges associated with these social factors. A recent study by Lieberz et al. has explored the distinctive characteristics of loneliness compared with social anxiety, and has concluded that lonely individuals have distinct behavioral and neural responsiveness features from those with social anxiety, particularly regarding decision-making and providing feedback in social settings.^24^ MI may be a low-cost intervention approach to improving healthy relationships.

MI intervention, when introduced before cognitive-behavioral therapy (CBT), has shown positive outcomes in patients with SAD. Romano et al. have proposed a casual model and studied a technical hypothesis (suggesting technical and relational factors may improve MI) in pre-CBT MI (n = 85) and identified that consistent therapist MI behaviors significantly predict the proportion of client change talk (client's statements' on willingness, potential/capability, reasons and requirement for change) in SAD.^25^ In an RCT published in 2019, an MI-based intervention (three sessions) given 12 weeks before CBT resulted in significantly better clinician-rated outcomes regarding the severity of social anxiety in patients with confirmed SAD who had high functional impairment.^26^ In the MIPAM trial, community-dwelling older adults randomized to PA monitoring alongside behavioral change intervention with MI (n = 32) had UCLA Loneliness Scale scores 2.3 points lower than those with PA monitoring alone (95% CI = −4.5, −1.24).^27^ Notably, MI has been reported to be effective in improving features of social dimensions in cancer settings and managing other chronic diseases. In a quasi-experimental study assessing emotional and instrumental aspects of the Social Support Scale, an MI intervention (four sessions/week; 30 min each) has been associated with higher scores among patients with breast cancer (n = 18).^28^ In the MOTIVATE-HF RCT, MI has been shown to help patients with heart failure improve their disease-specific QoL scores, as measured with the Kansas City Cardiomyopathy Questionnaire, at 3 (95% CI = 1.76, 11.71; d = 6.73) and 6 months (95% CI = 2.98, 13.84; d = 8.41) post-intervention. A longitudinal analysis demonstrated that an MI intervention among patients and caregivers resulted in significantly greater improvement than observed in the control group (95% CI = 0.26, 2.89; β = 1.57; p = 0.02).^29^ Moreover, in patients with stroke in a 2021 meta-analysis, MI had benefits in improving depression (p < 0.00001) and QoL (p = 0.0007) after 12 months of follow-up.^30^ Furthermore, in patients with HIV (n = 100), telephone-based MI interventions (one and four sessions) targeting risky sexual behavior resulted in lower depression, anxiety, and stress after 6 months.^31^

### Physical activity

Globally, a substantial proportion (7.2%) of deaths is attributable to PI; the proportions of attributable NCDs vary from 1.6% (hypertension) to 8.1% (dementia). PI-induced mortality rates show geographical variations among low- (4%), middle- (7%), and high-income (9%) countries.^32^ The COVID-19 pandemic has further negatively affected PA. A rapid review of 61 studies has indicated decreased levels of walking, bicycling, and mobility among people after than before the COVID-19 pandemic.^33^ Hence, campaigns are critical to promote WHO-recommended PA activity levels (150–300 min/week of moderate PA or 75–150 min/week of vigorous PA).^34^ MI may provide a promising tool to implement crucial steps progressively. MI intervention has been demonstrated to significantly increase in PA, improve psychological outcomes, and provide an excellent return on investment estimates.^35^

Several RCTs have indicated the effectiveness of MI in increasing PA among general and patient populations. The use of technology, such as mobile, telephone, or web-based approaches in MI, has been demonstrated to provide satisfactory outcomes.^36^^,^^37^ The investigators of the BEHOLD-16 RCT have pilot tested the feasibility and acceptability of a 16-week telephone-based combined positive psychology-MI intervention to promote PA in patients with T2DM. An average of 11 (of 14) sessions were completed by the participants, and a satisfactory composite mean score of ease/utility (8.6/10) was found. Investigators aim to explore the effects of MI on PA and clinical outcomes during the next stages of this trial.^38^ In an RCT of 161 children (age range: 9–16 yr) from Hong Kong who survived cancer, MI intervention delivered through mobile instant messaging has been found to result in a higher mean PA level (SD = 4.2) than that in controls over 1 year. The increased PA levels (intervention: 72.8% vs. control: 6.3%) also helped the children alleviate cancer- or treatment-associated adverse effects.^37^ In another trial of 334 primary-care patients, a 6-month face-to-face MI intervention (n = 203) resulted in a sustained improvement in walking at 6 (p = 0.006, d = 0.24) and 18 months (p = 0.032, d = 0.20), with respect to baseline.^39^ Moreover, the intervention group demonstrated a significant decrease in cholesterol levels at both the time points (p = 0.005, d = 0.31; p = 0.003, d = 0.33, respectively), with respect to baseline.^39^

In an RCT by Nasstasia et al., a combined 12-week MI-exercise intervention among 34 patients with major depressive disorder yielded promising outcomes in Beck Depression Inventory-II total score and factorial symptom subscales, and significant differential improvements in exercise participation were observed.^40^ In the MIPAM trial, the MI + PA monitoring intervention group walked 909 more steps daily than those with PA monitoring alone (95% CI = −71, 1889).^27^ Collins et al. have reported that MI increases walking distance at 3 months (40.5 m; 95% CI = 6.77, 61.34; p = 0.02) in patients with overweight/obesity and peripheral artery disease.^41^

Participant drop-out is a major concern in MI-based PA interventions. Wade et al. have identified several key indicators that may be reasons for drop-out.^42^ In a year-long community-based PA program of 619 participants in the United Kingdom (UK), 44.7% dropped out before week 12. Age, PA, musculoskeletal disorders, and endocrine system disorders have been found to be significantly associated with participant dropout.^42^

## Minimizing harmful substances

The risks caused by harmful substances and toxic habits, such as alcohol, smoking, excessive internet or social media use, gambling, recreational drugs, and excessive or inappropriate use of prescription drugs, are detrimental and are among the primary causes of preventable deaths.^4^ In 2020, tobacco smoking was prevalent in 32.6% (range: 32.2–33.1) of men and 6.5% (range: 6.3–6.7) of women worldwide, thus resulting in 7.0 (range: 2.0–11.2) million deaths.^43^ Harmful use of alcohol causes 3 million deaths annually and is attributable to 5.1% of the global burden of disease and injury.^44^ In the US, 0.5% of adults experience gambling disorder.^45^ Notably, COVID-19 has significantly affected gambling and alcohol consumption.^46^ Global action plans are critical to curtail these habits.

A recent cost-effectiveness analysis on MI has predicted 6-month incremental cost-effectiveness ratios of $1207–$1523 and $1040–$1313 per patient abstaining from unhealthful drinking and cannabis consumption, respectively.^47^ Several notable studies worldwide have demonstrated positive outcomes of MI in minimizing harmful substances. MI has been found to decrease alcohol consumption (RR = 0.49; 95% CI = 0.25, 0.95) among veterans with alcohol use disorder (n = 118), with a decline in drug use days at 1- and 3-month follow-up.^48^ In a Brazilian community-based cluster trial published in 2021, the smoking cessation rate in the MI group was 61.8% after 4 weeks of intervention, whereas that in controls was 47.7% (RR = 1.25; 95% CI = 1.01, 1.54; p = 0.043).^49^ In a 2017 study from Qazvin, Iran, group MI sessions (n = 8; 1 h each; twice weekly over 1 month) decreased the desire to use drugs (81.1%; p < 0.001) and probability of using drugs (81.9%; p < 0.001) among female drug users compared with controls.^50^ A recent exploratory qualitative study has assessed the mental health attitudes of emergency military professionals and veterans after 75-min MI sessions. The interviews focused on mental health, substance use, and identity development, and yielded favorable results regarding self-stigma barriers relevant to help-seeking.^51^

## Sleep

Sound sleep of 7–9 h nightly is critical for self-care, because it is associated with building long-term immunity; maintaining good cognitive function and mental wellbeing; overcoming stress and memory decline; reducing the risk of anxiety, depression, and post-traumatic stress disorder; limiting the long-term effects of loss and grief; supporting effective functioning of the microbiome and the body's metabolism; and preventing the risk of obesity, T2DM, high blood pressure, and ischemic heart disease.^4^ A 2019 global literature-based analysis has estimated that 936 (95% CI = 903, 970) and 425 (95% CI = 399, 450) million adults (age range: 30–69 yr) experience mild-to-severe and moderate-to-severe obstructive sleep apnea (OSA), respectively, and high prevalence is observed in China, followed by the US, Brazil, and India.^52^ A US-based large-scale population analysis has identified that drivers with OSA have 17% greater risk of experiencing a motor vehicle accident than those without OSA (adjusted HR = 1.17; 95% CI = 1.13, 1.20).^53^

Researchers worldwide have documented favorable results of using MI interventions in managing sleep disorders across various settings. Continuous positive airway pressure (CPAP) is the standard of care for patients with sleep apnea, and treatment non-adherence is a known problem. MI interventions help improve adherence. In an RCT of patients with OSA, a multidimensional intervention comprising semi-structured MI (n = 28) resulted in significantly greater mean daily use of PAP (p = 0.03) than that in controls.^54^ The results of the MEntA RCT have provided strong evidence supporting the application of MI in improving CPAP adherence. MI significantly improved adherence (mean difference = 1.60 h; 95% CI = 0.60, 2.61; p < 0.01) and was associated with statistically significant changes in the Questionnaire of Evaluation of Perceived Competence in Adherence to CPAP in OSA (mean difference = 4.61; 95% CI = 3.49, 5.72; p < 0.001) and QoL (p < 0.001).^55^ An ongoing multicenter RCT, the MotivAir study, is assessing the effectiveness of a telephone-based MI intervention in improving CPAP adherence among patients with OSA. Further studies should be valuable in increasing understanding of how MI intervention increases CPAP adherence and the Apnea-Hypopnea Index, as well as secondary outcomes, such as motivation, perceived competence, QoL, and sleepiness at various intervals (1-, 3-, and 6-months).^56^

In a Chinese RCT in patients with obesity (n = 100), MI intervention significantly improved sleep status and decreased the Epworth Sleepiness Scale and Self-Rating Scale of Sleep scores (p < 0.05).^57^ In patients who underwent thoracolumbar spine surgery (n = 15), MI played a crucial role in significantly improving confidence levels, as measured with the Health Confidence Index with self-care management of symptom-associated disability, including lack of sleep (p = 0.002).^58^ In another study, Zaslavsky et al. have pilot tested the feasibility and efficacy of wearable technology and MI in improving sleep in older patients with osteoarthritis and sleep disturbance (n = 24; mean age: 71 yr). The Insomnia Severity Index and Acceptance of Sleep Difficulties scores improved 1.2 (95% CI = −2.43, −0.05; p = 0.04) and 2.5 (95% CI = 0.9, 4.9; p = 0.02) points, respectively, over 19 weeks.^59^ Chronic sleep deprivation is a common health issue among schoolchildren. Early school schedules across countries affect childrenin addition to natural delay of circadian rhythms. In a recent RCT, adolescents (mean age: 15.8 ± 0.98 yr) who took part in an MI intervention (four weekly group sessions and text reminders for 3 weeks) (n = 212) reported earlier bedtimes during school days (p = 0.004), greater intention to make behavioral changes (p = 0.043), increased sleep duration (p = 0.089), and lower levels of daytime sleepiness (p = 0.001).^60^

## MI training for healthcare professionals and students

**Figure 1:**
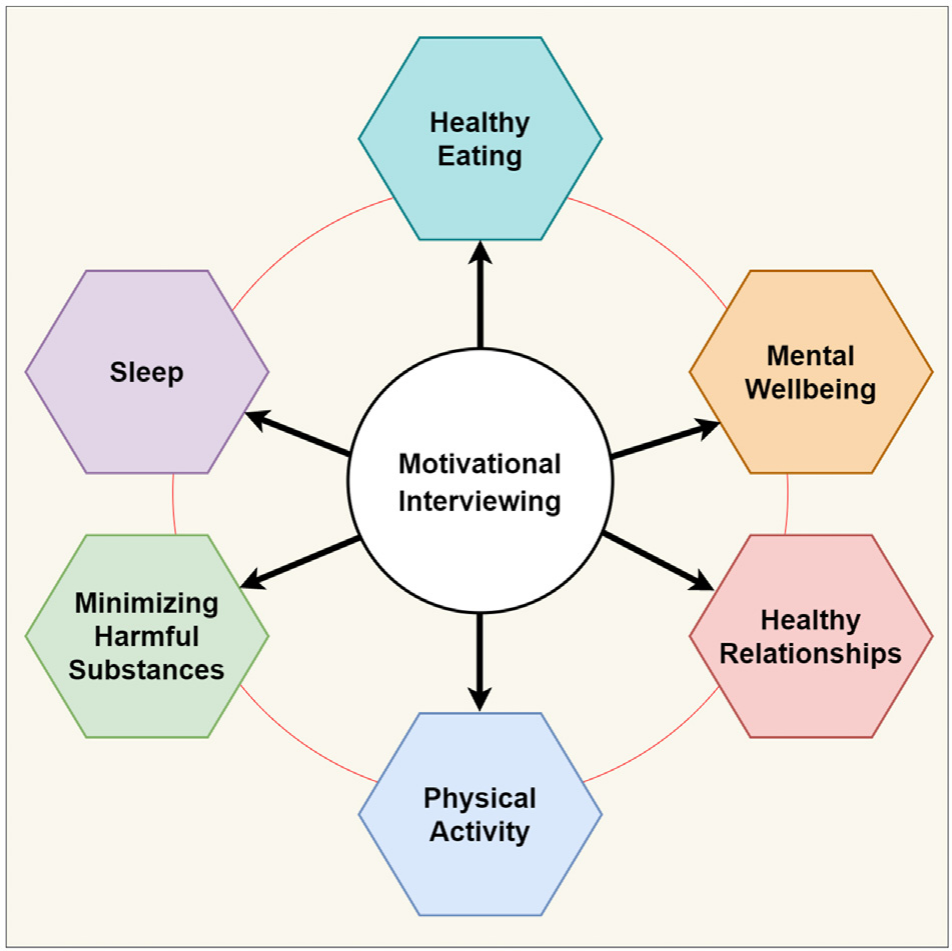
Application of MI in the six pillars of LSM.

Extensive literature evidence has indicated the benefits of MI treatment, and interest in research on MI application is increasing across all BSLM pillars (Figure 1). However, MI poses potential challenges. For effective outcomes, MI interventions require time to develop relationships and trust with patients, cognitive clarity, and demand motivation. It works well when proper follow-up sessions are conducted.^61^ Sannes has described three barriers to MI: provider barriers (knowledge, attitude, skills, and behavioral routines), client/patient barriers (knowledge, attitude, skills, and adherence), and practice barriers (organization, resources, and structures).^62^

As of November 8, 2022, 1474 studies on MI have been registered at ClinicalTrials.gov.^63^ Even interventions of shorter duration have been reported to yield better outcomes.^64^ MI assists patients in relating their current behavior and emotions to the adverse outcomes that could occur if they do not implement changes. Clinicians and other healthcare professionals must understand the relevance and significance of MI in clinical practice.

McKenzie et al. have analyzed video recordings of consultations with general practitioners, coded with the MI Treatment Integrity coding system (n = 60) (n = 32) across 16 practices in Glasgow, Scotland. Simple and complex reflections were observed in 67% and 28% of consultations, respectively. Notably, no physicians met beginner-level proficiency requirements for the technical global rating of actions taken to inspire patients toward behavior change. Furthermore, 18% of consultations showed a confrontation—an MI-inconsistent approach.^65^ Hence, comprehensive training on MI among healthcare professionals may help achieve and sustain positive changes. Tsai et al. have pilot tested the effectiveness of MI training (2-day workshop, followed by 2 monthly sessions; 55 sessions total) for mental illness peer specialists. The MI Inconsistent Adherence Scale and Sharing Lived Experience Adherence Items scores decreased at the 3-month follow-up, and reductions in offering unsought guidance and stressing absolute abstinence were observed.^66^ In another pilot study, rehabilitation nurses in the UK were trained in MI, and quantitative and qualitative measures were assessed after 2 and 8 months. The authors concluded that conducting MI training for nurses is feasible and appropriate to their profession.^67^ In 2021, Lozano et al. validated an MI intervention for inclusion in the Iowa State Nursing Interventions Classification and the Intervention Normalization for Nursing Practice projects.^68^ An Australian study has reported the benefits of conducting MI training for nurses. The participants gained improvements in knowledge and confidence scores with a proper understanding of MI principles and strategies.^69^

Health workforce shortages are a global concern; students in the healthcare academic domains contribute to this demand. MI interventions could also be led by students after adequate training. In a Canadian medical school, students (n = 27) reported a significant increase in MI knowledge after attending an educational workshop on MI (p = 0.001).^70^ Notably, a student-led MI program to promote PA levels has helped rural Australian adults: 98% of interviewees found the intervention meaningful, whereas 96%, 88%, and 98% of participants reported that the session was empathetic, was autonomy-focused, and helped them develop sustained behavior change, respectively.^71^ Similar research on MI training has been conducted among students of nutrition, physiotherapy, occupational therapy, and sport and exercise science.^72^, ^73^, ^74^

## Conclusion

Lifestyle change is often a gradual process involving multiple efforts and setbacks. MI works on the basis the idea that change is a process, not an event. This interdisciplinary, whole-system approach helps address the known issue of uncertainty regarding change. MI serves people to achieve sustainable lifestyle changes and helps avoid the threat of NCDs. The roles of MI practitioners are critical in obtaining satisfactory outcomes. Thus, expert practitioners are expected to commit time and effort, and to be interested in increasing skills and gaining methodological knowledge of this interdisciplinary approach.

## Source of funding

This research did not receive any specific grant from funding agencies in the public, commercial, or not-for-profit sectors.

## Conflict of interest

The authors have no conflict of interest to declare.

## Ethical approval/consent

Not applicable.

## Authors’ contributions

MA conceived the review's idea. SI and HI performed the literature review. MA wrote the initial draft. HI edited the manuscript substantially. All authors reviewed and agreed to the final version. All authors have critically reviewed and approved the final draft and are responsible for the content and similarity index of the manuscript
